# Neuroenhancement of Memory for Children with Autism by a Mind–Body Exercise

**DOI:** 10.3389/fpsyg.2015.01893

**Published:** 2015-12-11

**Authors:** Agnes S. Chan, Yvonne M. Y. Han, Sophia L. Sze, Eliza M. Lau

**Affiliations:** ^1^Neuropsychology Laboratory, Department of Psychology, The Chinese University of Hong KongHong Kong, China; ^2^Chanwuyi Research Center for Neuropsychological Well-Being, The Chinese University of Hong KongHong Kong, China; ^3^Henan Songshan Research Institute for ChanwuyiHenan, China; ^4^Department of Rehabilitation Sciences, The Hong Kong Polytechnic UniversityHong Kong, China

**Keywords:** neurocognitive enhancement, memory, functional connectivity, mind–body training, autism, EEG

## Abstract

The memory deficits found in individuals with autism spectrum disorder (ASD) may be caused by the lack of an effective strategy to aid memory. The executive control of memory processing is mediated largely by the timely coupling between frontal and posterior brain regions. The present study aimed to explore the potential effect of a Chinese mind–body exercise, namely *Nei Gong*, for enhancing learning and memory in children with ASD, and the possible neural basis of the improvement. Sixty-six children with ASD were randomly assigned to groups receiving *Nei Gong* training (NGT), progressive muscle relaxation (PMR) training, or no training for 1 month. Before and after training, the participants were tested individually on a computerized visual memory task while EEG signals were acquired during the memory encoding phase. Children in the NGT group demonstrated significantly enhanced memory performance and more effective use of a memory strategy, which was not observed in the other two groups. Furthermore, the improved memory after NGT was consistent with findings of elevated EEG theta coherence between frontal and posterior brain regions, a measure of functional coupling. The scalp EEG signals were localized by the standardized low resolution brain electromagnetic tomography method and found to originate from a neural network that promotes effective memory processing, including the prefrontal cortex, the parietal cortex, and the medial and inferior temporal cortex. This alteration in neural processing was not found in children receiving PMR or in those who received no training. The present findings suggest that the mind–body exercise program may have the potential effect on modulating neural functional connectivity underlying memory processing and hence enhance memory functions in individuals with autism.

## Introduction

Neurocognitive enhancement can be broadly defined as any method/application, be it pharmacological or non-pharmacological based, that can enhance cognitive functions with some neuroscience basis. The feasibility of neurocognitive enhancement relies on the basic assumption that the brain is plastic for change and its function can be altered. Some neurocognitive enhancement approaches utilize specific techniques such as transcranial magnetic stimulation (TMS; Solé-Padullés et al., 2006; Luber and Lisanby, 2014) and transcranial direct current stimulation (tDCS; Clark and Parasuraman, 2014; Coffman et al., 2014). Others are more behavioral-based, such as learning a musical instrument (Chan et al., 1998; Roden et al., 2012), mindfulness training (Chiesa et al., 2011), or exercises (Smith et al., 2010; Roig et al., 2013). Positive findings were reported on improving learning and memory with various neurocognitive enhancement methods including tDCS in healthy adults (Marshall et al., 2004; Hammer et al., 2011; Floel et al., 2012) and patients with Alzheimer’s disease (Boggio et al., 2009, 2012), mindfulness training in healthy individuals and in formerly depressed patients (Heeren et al., 2009; Hargus et al., 2010), cardiovascular exercise in young adults to old aged people (Chan et al., 2005; Smith et al., 2010; Roig et al., 2013). Furthermore, regular practice of mind–body exercises have been shown to improve cognitive function and memory performance on elderly with and without cognitive impairments (Chan et al., 2005, 2014b; Lam et al., 2012; Wayne et al., 2014). However, in spite of the encouraging empirical evidence for the treatment efficacy of neuroenhancement techniques on memory and learning, most previous studies were done on adults and the effects and applicability of neuroenhancement approaches such as mind–body intervention on the pediatric population was not well-known.

Autism spectrum disorders is a group of lifelong developmental disorders characterized by poor social skills, language impairment, and abnormal repertoire of stereotyped behaviors. Abnormalities are also found in higher cortical functions, such as memory. To date, there is no known cure for autism. Early intervention, however, has been found to be very important in improving the subsequent functioning of these children. Given the potential beneficial effects of neurocognitive enhancement techniques on memory (Chan et al., 2005, 2014b; Chiesa et al., 2011; Boggio et al., 2012; Floel et al., 2012), the present study aims to explore if practicing a Chinese mind–body exercise, namely “*Nei Gong*,” can help enhancing memory processing in children with ASD. The *Nei Gong* is developed based on doctrines of *Chanwuyi* (i.e., Zen, martial arts, and healing) that originated from the *Shaolin* Temple (Chan, 2013; Chan and Sze, 2013). Recent empirical evidence has reported its effects on altering the neuroplasticity in terms of electrophysiological activity and connectivity patterns of brain at resting state or during a cognitive task after regular practice of the *Nei Gong* for a certain period of time (e.g., Chan et al., 2011b, 2012, 2013a). A randomized controlled trial (RCT) study has revealed increased electroencephalographic (EEG) frontal alpha asymmetry index (a measure associates with positive mood and relaxation) and frontoposterior theta coherence (a measure associates with attentiveness) in community-dwelling adults after 1 month of *Nei Gong* practice (Chan et al., 2011a).

Furthermore, a recently published RCT on 46 children with ASD revealed that 1 month of *Nei Gong* practice induced significant improvement in inhibitory control ability of ASD children which coincided with their elevated EEG source activity in the anterior cingulate cortex (Chan et al., 2013b). In another study conducted by Chan et al. (2014b), a group of elderly people with lower memory function showed a 50% enhancement in memory recall performance after 3 months of mind–body training. A child with autism and severely impaired memory functioning was able to achieve a low-average to average level of total recall after receiving 1 month of training (Chan et al., 2011d). Given the abovementioned encouraging findings, the present study aimed to further examine the potential of *Nei Gong* as a possible neurocognitive enhancement on memory functions of children, and anticipated that 1 month of *Nei Gong* practice may enhance memory processing and change the neuroelectrophysiological activity of the brain in ASD.

While some individuals with ASD show severely impaired memory, some fall into the other extreme category, with ‘savant’ memory (O’Connor and Hermelin, 1989; Mottron et al., 1998). Although the exact memory profile remains unclear, empirical evidence has suggested that the memory problems of individuals with ASD are associated with their executive dysfunction, resulting in the ineffective use of strategies to monitor, organize, and maintain to-be-learned material. Our previous studies on memory functions in children with ASD also revealed a significantly lower total recall of newly learned information and less effective utilization of strategies to facilitate memory compared with typically developing children (Cheung et al., 2010; Chan et al., 2011c). The ineffective use of strategies to integrate information across contexts, resulting from deficient executive function, has been hypothesized as one of the causes of memory deficits in individuals with ASD. The extent of memory deficits in ASD is more severe when cognitive tasks require significant mental effort or when information is meaningful, semantically related or in vast amounts (Minshew and Goldstein, 1993, 1998, 2001; Minshew et al., 1997; Toichi and Kamio, 2002, 2003; Williams et al., 2006). Provision of explicit semantic cues during encoding and recall can improve memory performance in individuals with ASD (Tager-Flusberg, 1991; Mottron et al., 2001), which further supports the role of executive dysfunction in the memory deficits associated with ASD.

The executive control of memory processing that is mediated by the prefrontal cortex and its distributed network in the posterior cortical regions (particularly the posterior association cortex in the parietal lobe; Weiss et al., 2000; Fletcher and Henson, 2001; Rugg et al., 2002; Nyberg et al., 2003; Osaka et al., 2004; Sauseng et al., 2005). Although the memory profile in ASD remains uncertain, and little is known regarding the underlying basis of the deficits, it has been suggested that abnormalities in cortical connectivity between key neural networks in the frontal and posterior brain regions may account for the dysexecutive control of memory processing associated with the disorder (Weiss and Rappelsberger, 2000; Fletcher and Henson, 2001; Rippon et al., 2007; Chan et al., 2011c). Evidence for disordered connectivity across neural systems in ASD patients was found in diffusion tensor imaging (DTI) studies that showed reduced myelin integrity in the ventromedial prefrontal cortex and at the temporoparietal junctions in individuals with ASD (Barnea-Goraly et al., 2004; Lewis and Elman, 2008). Furthermore, electrophysiological studies in children with ASD using coherence, a quantitative EEG measure that estimates the level of synchrony or cortical connectivity between brain areas in response to cognitive processes (Thatcher et al., 2005a; Rippon et al., 2007; Coben et al., 2008), also showed disordered connectivity between the frontal and posterior brain areas that was associated with executive dysfunction (Han et al., 2013) and memory deficits (Chan et al., 2011c). In addition, there is evidence from different coherence measures to suggest that coherence in the theta band (4–7.5 Hz) is related to the executive control of memory processes (Sauseng et al., 2005; Mizuhara and Yamaguchi, 2007). Specifically, evidence from different coherence measures has indicated that greater EEG coherence in the theta band between the frontal lobe and its posterior networks is associated with the successful encoding and storage of episodic information (Volf and Razumnikova, 1999; Weiss and Rappelsberger, 2000) as well as with success in memory tasks that involve manipulation rather than simple retrieval and maintenance of information (Sauseng et al., 2005). These findings support the idea that effective memory processing depends on the timely activation of cortical areas, which is measured by a higher level of EEG theta coherence. The memory deficits in individuals with ASD may be the result of poor orchestration between different brain areas due to aberrant connectivity that interrupts efficient cognitive processing.

In view of the ineffective use of memory strategies and its associated pathological functional connectivity in ASD, we thus attempt to explore the effect of *Nei Gong* practice on improving the executive control of memory process, in terms of organizational strategies, in the children with ASD, and modulating underlying neural synchronization using EEG theta coherence when the ASD children were performing a memory task. We anticipated that *Nei Gong* would foster more effective strategies for new learning and hence resulting in better memory performance in children with ASD. Also, the memory-enhancing effect would coincide with a more synchronized EEG coherence pattern in the neural networks connecting the frontal and posterior brain regions.

## Materials and Methods

### Participants

Sixty-six children with ASD between 5 and 17 years of age voluntarily participated in the study with their parents’ written consent. The children were recruited from three primary schools and one secondary school in Hong Kong and the existing database at the Neuropsychology Laboratory of the Chinese University of Hong Kong. All participants received a formal diagnosis of Autistic Disorder or Pervasive Developmental Disorders Not Otherwise Specified (PDD-NOS) by a clinical psychologist through a standard clinical interview with their parents based on DSM-IV-TR criteria (American Psychiatric Association [APA], 2000). The clinical psychologist also assessed the severity of each child’s autistic symptoms using the autism diagnostic interview-revised (ADI-R; Lord et al., 1994). The interview included detailed questions regarding the child’s early development and current functioning, with higher scores indicating more severe autistic symptoms. Children with other neurodevelopmental, psychiatric or neurological comorbidities and children who were taking prescribed psychiatric medication were excluded from the study.

The children were randomly assigned into one of the three groups: (1) NGT; (2) progressive muscle relaxation (PMR) training; and (3) no training control. Of the 66 children, 11 failed to complete the memory task due to limited comprehension ability or non-compliant behaviors, and seven withdrew from the study before the post-intervention assessment due to personal reasons. The remaining total number of participants in the NGT, PMR and control groups was 18, 17 and 13, respectively. There was no significant difference in attrition rate among groups [χ^2^(2) = 3.21, *p* = 0.20]. **Table 1** presents the demographic and clinical characteristics, and the attrition rate of each group. The three groups were matched for age [*F*(2,45) = 1.49, *p* = 0.24], gender [χ^2^(2) = 0.20, *p* = 0.90], level of intelligence [*F*(2,45) = 1.24, *p* = 0.30], diagnosis based on DSM-IV-TR criteria [χ^2^(2) = 4.55, *p* = 0.10], and severity of autistic features as measured by the four ADI-R subscales, with *F* ranging from 0.14 to 1.22, and *p* ranging from 0.22 to 0.87. The level of intelligence of each child was assessed by a research assistant using the short form of the Chinese version of the Wechsler Intelligence Scale for Children-Fourth Edition [Hong Kong; WISC-IV(HK); Wechsler, 2010] or the Stanford–Binet Intelligence Scale-Fourth Edition (SB-FE; Thorndike et al., 1986) for non-verbal children and those who showed a floor effect in the WISC-IV(HK). Six children in the NGT group, three children in the PMR group and five children in control group had limited intelligence, with an IQ score below 70.

**Table 1:** Baseline demographic and clinical characteristics of each group.

Characteristic	Control (*n* = 13)	PMR (*n* = 17)	NGT (*n* = 18)	*F* or χ^2^	*p*
Age, years	9.61 (3.35)	11.04 (3.33)	11.88 (4.07)	1.49	0.24
Gender-male (%)	92.3	88.2	94.4	0.20	0.90
IQ	85.92 (29.25)	86.53 (17.46)	76.28 (17.71)	1.24	0.30
Diagnosis					
Autistic disorder (%)	100	88.2	72.2	4.55	0.10
PDD-NOS (%)	0	11.8	27.8		
Severity of disorder					
ADI-R social interaction	20.15 (6.50)	23.06 (7.05)	23.39 (4.67)	1.22	0.31
ADI-R communication	20.77 (4.38)	17.59 (5.78)	18.94 (4.33)	1.55	0.22
ADI-R stereotyped behavior	7.00 (2.86)	6.53 (2.81)	6.50 (2.90)	0.14	0.87
ADI-R Abnormal < 36 months	2.92 (1.32)	2.94 (1.78)	3.78 (1.70)	1.49	0.24
Attrition rate (%)	40.9	22.7	18.2	3.21	0.20

*Control, control group without training; PMR, progressive muscle relaxation; NGT, *Nei Gong* training; ADI-R, autism diagnostic interview-revised; IQ, intelligence quotient as assessed by the Chinese version of the Wechsler Intelligence Scale for Children-Fourth Edition (Hong Kong) or the Stanford-Binet Intelligence Scale-Fourth Edition; PDD-NOS, pervasive developmental disorders not otherwise specified. Standard deviations are in parenthesis.*

### Ethics

This study was conducted in accordance with the Helsinki Declaration of the World Medical Association Assembly. The research protocol was approved by the Human Subjects Ethics Sub-committee (HSESC) of the Hong Kong Polytechnic University (Ref. No. HSEARS20120517001) and the Chinese Clinical Trial Registry (Registration No. ChiCTR-TRC-12002561).

### Procedure

**FIGURE 1:**
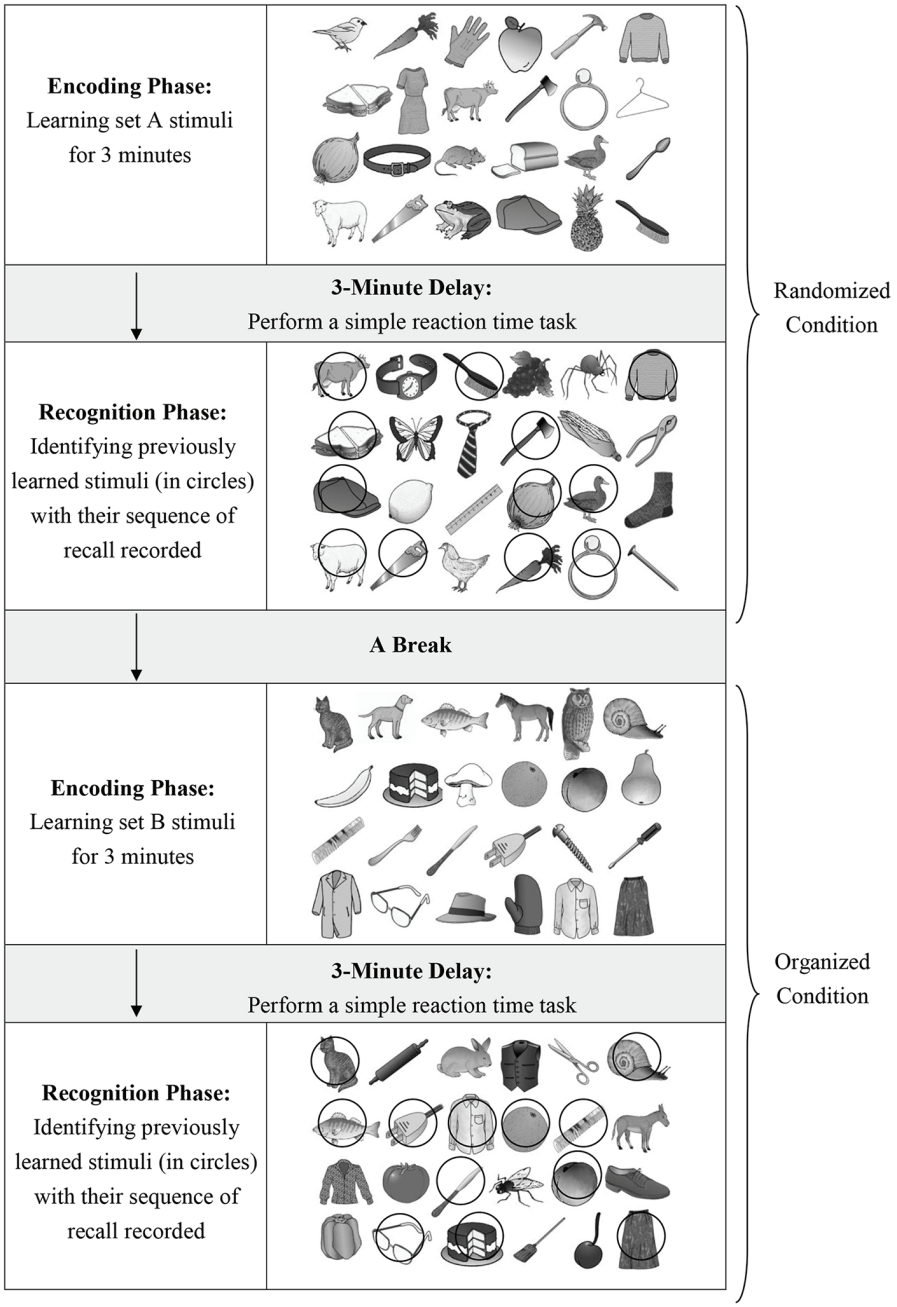
**An illustration of the experimental paradigm of the visual memory task**.

Prior to the baseline assessment, the children and their parents were briefed on the assessment procedure, and informed consent was obtained from the parents. The children were individually assessed for their intellectual functioning and scalp EEG activities were recorded in a quiet room by trained research assistants. During the assessment, the children’s parents were interviewed by a clinical psychologist regarding the child’s developmental and medical histories using a structured clinical interview. The clinical psychologist and research assistants who conducted the assessments were blinded to the rationale of the study and the group assignment. During the EEG recording session, each child was required to perform a visual memory task while their EEG data were acquired at 256 Hz using a Deymed TruScan 32 EEG System with 19 electrodes positioned across the scalp with an electrode cap according to the International 10–20 System (Klem et al., 1999). The electrode impedances were maintained at ≤10 kΩ. The EEG signals were referenced to linked ears with band-pass filtered in the range of 1–40 Hz. EEG was collected after presentation of the Learning set stimuli for 3 min during the encoding phase (**Figure 1**) when the child performed the visual memory task (see Visual Memory Task). Throughout the EEG recording session, the child was alerted by an experienced research assistant upon any signs of reduced vigilance, and body movements were time-marked for off-line analyses. EEG data were stored and later display on a computer for selection and analysis. Artifact-free EEG data were selected based on both computer selection (reject levels were set at ±150 μV) and visual examination for eye movements and muscle artifacts using the NeuroGuide software program. A minimum of 1 min of artifact-free data were randomly selected throughout the 3-min data (John et al., 1988 for discussion of qEEG method) with each segment lasting for at least 600 ms according to the NeuroGuide software manual. All selected artifact-free EEG data were then spliced together as a series of continuous 256 digital values with epoch length of 2 s. The splice artifact was minimized and the EEG segments were processed with sliding average of overlapping fast Fourier transformed (FFT) windows (75% overlap) using the NeuroGuide software. The FFT EEG data were then captured for subsequent coherence and standardized low resolution brain electromagnetic tomography (sLORETA) analyses. Theta coherence measures (4–7.5 Hz) were used in the present study and computed from the average of single trials.

After the baseline assessment, the two training groups were provided with NGT and PMR for 1 month, while the other group of children who was not provided with any of the two trainings during the 1-month period served as a control. The same set of assessments of visual memory and EEG activity was administered to the children after training.

### Training Program

The *Nei Gong* and PMR training were conducted at the Chinese University of Hong Kong twice per week for 4 weeks, with each session lasting an hour. The training programs were developed based on the approach adapted from Greenhalgh (1997) in order to protect against sources of bias. Children were randomly allocated to the treatment and control groups to avoid selection bias. To minimize performance bias and bias results from clinician’s personal preference or expertise in one training method over another, both training programs followed standard protocols, and were taught by highly experienced clinical psychologists with over 10 years of clinical experience in treating children with autism. The training programs were administered on the same days of the week in the same classroom. Furthermore, to prevent potential detection or evaluation bias, the instructors of the *Nei Gong* and PMR training were not involved in the recruitment, consent, group allocation, or assessment procedures of the participants. **Table 2** has summarized the differences between PMR and NGT.

**Table 2:** Comparison between progressive muscle relaxation and *Nei Gong* training.

	PMR	NGT
Origin	A well-documented component of cognitive behavioral therapy developed in the Western countries	Developed based on a traditional Chinese mind–body practice, namely *Chanwuyi*
Form of practice	Systematic contraction and relaxation of seven muscle groups from head to feet in a fixed sequence following the voice instruction	Sets of slow, gentle, and simple bodily movements that can be practiced with specific pieces of music
Duration of practice	•Each round of practice lasts for 20 min	•No restriction on the duration
	•Recommend to practice once per day	•Recommend to practice everyday and to stop when beginning to sweat to avoid exhaustion
Potential benefits	•Attain mental calmness	•Foster self-awareness and self-control
	•Reduce anxiety	•Help restore calmness and relaxation
	•Reduce behavioral disturbances	•Reduce stress
		•Increase flexibility of limbs
		•Improve blood and *Qi* circulation

### Nei Gong

The children in the NGT group were taught *Nei Gong* by a clinical psychologist who was highly experienced with the application of NGT for children with special needs. *Nei Gong*, which was developed based on the *Chan* medical model, are sets of slow movements that emphasize smooth, gentle and calm movements, and the maintenance of a natural and relaxed attitude during practice. The major functions of *Nei Gong* include fostering self-awareness and mental self-control to help restore a calm and relaxed state, reducing stress, and increasing flexibility of the limbs. A fundamental Chinese medical concept suggests that gathering and filling the *Qi* (bioenergy) inside the body is a way to maintain mental and physical health. *Nei Gong*, when practiced in a natural and relaxed manner, can facilitate the circulation of *Qi* and blood. Furthermore, regular practice of *Nei Gong* has been found to help fostering a more efficient brain as supported by some EEG and fMRI data. Our previous randomized controlled study revealed that constant practice of *Nei Gong* was able to foster a simultaneously relaxed and attentive brain state, as reflected by increased EEG alpha asymmetry and intra- and inter-hemispheric EEG theta coherence indices (Chan et al., 2011a). The co-existence of a relaxed and an attentive mind is crucial for achieving peak performance (Norris and Currieri, 1999; Ros et al., 2009). Some unpublished fMRI data from four practitioners of *Nei Gong* also showed higher activation of frontal and temporal brain regions and some limbic brain structures that are crucial for mediating attentional, memory and emotional processing, while they were practicing the *Nei Gong.* Detailed descriptions and demonstration of the five movements can be found in Chan et al. (2013b) and http://www.chanwuyicenter.com. Five forms of *Nei Gong* movements were selected, arranged in a fixed sequence and incorporated with specific pieces of music to facilitate the children’s mastery of the technique and to keep them engaged. While practicing *Nei Gong*, the children were guided to move calmly and in a relaxed manner and were encouraged to persist with the movements. The selected *Nei Gong* movements involved simple bodily actions (e.g., moving hands/fingers up and down, and bending the knees), which can be easily mastered by children with moderate mental retardation. Each round of *Nei Gong* with music lasted for 5 min. The children were closely monitored by the clinical psychologist during each session.

The children were encouraged to practice the movements daily one to three times. The children’s practicing times and frequencies were recorded in log books, which indicated that 65% of the children practiced *Nei Gong* at least 6 days per week for between 5 and 45 min a day (mean = 19.38; *SD* = 11.82). The remaining children practiced 5–25 min a day, 3–5 days per week (mean = 13.66; *SD* = 6.12). Given that physical conditions varied among the children, they were instructed to halt the exercise if they began to sweat to avoid exhaustion. Thus, the practice duration was not fixed.

### Progressive Muscle Relaxation

The children in the PMR training group were taught the PMR technique by another clinical psychologist with expertise in administering PMR to the pediatric population. PMR was selected as a comparison to *Nei Gong* because it is a well-documented component of cognitive behavioral therapy, which has been reported to help children with ASD attain mental calmness and reduce anxiety and behavioral disturbances and because it is a form of exercise-like training involving the systematic relaxation of muscle groups (Reaven, 2009). As compared to *Nei Gong*, PMR serves similar purpose of helping one to relax and be calm, yet it probably has limited effect on self-control, limb flexibility, and blood and Qi circulation as *Nei Gong* does. The Chinese version of PMR for children was adopted. This version was locally developed by the Clinical Psychology Division of the Hong Kong Psychological Association and has been used in pediatric clinical practice since 2004. Clinical experience and empirical evidence (Omizo et al., 1982; Zipkin, 1985) have suggested that even children with mental retardation can master the PMR technique. In each training session, the therapist guided the children in sequentially tensing and relaxing seven muscle groups (nose, mouth, shoulders, arms, hands, chest, and feet), as instructed by a soundtrack. During the practice, visual and verbal cues were provided on how to contract and relax each muscle group (e.g., the tensing-relaxing of the arm muscle was represented by an image of a monkey swinging from one tree to another printed on a cue card together with the voice of a monkey presented on the sound track). The therapist monitored the progress of each child and observed that all children were able to master the technique. Each round of PMR lasted approximately 20 min.

After the first training session, the children were given a cue card that listed the seven steps of the PMR technique for home practice. In each subsequent training session, any difficulties the children encountered during their home practice sessions were reviewed. Half of the children practiced PMR once (i.e., 20 min) a day at least 6 days a week, while the other half practiced between 1 and 5 days per week. The average practice duration of the NGT and PMR groups was similar (*t* = -1.28, *p* = 0.21).

### Visual Memory Task

Electroencephalography signals for each child were collected during a visual memory task. This task was developed by the authors and consists of two sets of 24 line drawings taken from the Snodgrass and Vanderwart’s (1980) object database, modified and validated by Rossion and Pourtois (2004). This test was adopted in the present study as our previous study found significant impairments in memory recall in this visual memory task in children with ASD compared with age-matched normal controls (Chan et al., 2009, 2011c). The deficient memory performance in individuals with ASD was associated with abnormal neural connectivity (Chan et al., 2011c).

The two sets of line drawings (Set A and Set B) were placed in a six by four array displayed on a computer screen (see an example in **Figure 1**). The participant was asked to memorize and recall each set of line drawings one after one. Each set consisted of six line drawings belonging to each of the four semantic categories: animals, tools, food, and clothing. Each set of line drawings has two formats, i.e., randomized or organized, which indicates whether the line drawings are placed in random manner or organized according to their belonging category (see **Figure 1**). In the organized condition, the placement of line drawings was pre-organized such that items belonging to the same category were placed in the same row, which aims to provide an external organization cue to facilitate the learning process. Each participant was firstly presented with the randomized line drawings for studying for 3 min (i.e., the encoding phase in **Figure 1**), followed by a 3-min delay, and then was required to recognize as many as possible the 12 previously learned line drawings (i.e., the targets) from 12 new line drawings (i.e., the distracters). During the 3-min delay, the participant was required to engage in a simple reaction time task so as to keep him/her attended on a non-memory task. The simple reaction time required the participant to press a response key as quickly as possible when a black circle emerged in the center of the computer screen. During the recognition trial, the participant was given a response sheet on which 12 targets and 12 distracters were scattered, and he/she was asked to circle the targets that they remembered. The sequence in identifying each target was recorded manually by the examiner. No feedback on the correctness of response was given to the participant. After that, the organized form of another set of line drawings was presented to the same participant for encoding, which was followed by the delay task and then the recognition trial. Their EEG signals during the 3-min encoding phase of each set of stimuli were captured and spectrally processed for computation of the coherence data in the theta band (4–7.5 Hz) and localized for the sources of theta activity using the sLORETA method.

### Measures

#### Memory Function Measures

Three memory measures were adopted to assess the executive control of memory processing in ASD. The memory strategies adopted by each child to facilitate memory recall were reflected by the semantic clustering score and the visual scanning score, whereas the level of memory performance was reflected by the total recall score. These measures were obtained during both randomized and organized conditions, and the details of each measure are elaborated below.

#### Semantic Clustering Score

This is a measure of strategies adopted during memory retrieval. It is calculated as the number of target items belonging to the same semantic category that were consecutively identified by a child. The maximum score is 8. Higher scores indicate a higher tendency to use categorization methods in memory retrieval.

#### Visual Scanning Score

This is another measure of memory retrieval strategies. It is calculated as the number of target items consecutively identified by the child that were placed proximally to each other in the array. For instance, if the first identified item is located adjacent horizontally or vertically to the second identified item, then 1 mark will be scored. The maximum score is 11. Higher scores indicate a higher tendency to use a systematic visual scanning strategy during memory retrieval.

#### Total Recall Score

To measure the ability to retrieve newly learned information from memory during a recognition task, each correct identification of a previously learned item (i.e., the target items) was scored as 1. The maximum score of total recall was 12; higher scores indicate better memory recall.

### EEG Measures

#### Theta Coherence

Coherence, defined as the cross-spectral power between an electrode pair normalized by their power spectra, is an index that measures temporal synchronization of EEG activity between two brain regions underneath the electrodes and reflects the functional connectivity between the two regions (Sauseng et al., 2005; Coben et al., 2008). 171 coherence values were computed as the results of all combinations of couple among the 19 electrode positions (Fp1, Fp2, F3, F4, F7, F8, Fz, T3, T4, T5, T6, C3, C4, Cz, P3, P4, Pz, O1, and O2; i.e., n(n-1)/2 = 19^∗^18/2 = 171). Based on Sauseng et al. (2005) paradigm and results from our previous electrophysiological studies on memory deficits and executive dysfunctions in children with autism (Chan et al., 2011c; Han et al., 2013), two clusters of coherence measures within and between hemispheres were computed to examine the functional coupling between the EEG signals acquired from the frontal and posterior scalp regions during memory encoding under both randomized and organized conditions. There were eight mean coherence values, four intra-hemispheric coherence values (intra-frontal: F3–F7, F4–F8; intra-posterior: P3–O1, P4–O2; intra-left frontoposterior: F7–P3, F7–O1, F3–P3, F3–O1; intra-right frontoposterior: F8–P4, F8–O2, F4–P4, F4–O2); and four inter-hemispheric coherence values (inter-frontal: F3–F4, F7–F8, F3–F8, F4–F7; inter-posterior: P3–P4, O1–O2, P3–O2, P4–O1; inter-left-to-right frontoposterior: F7–P4, F7–O2, F3–P4, F3–O2; inter-right-to-left frontoposterior: F8–P3, F8–O1, F4–P3, and F4–O1).

#### Theta Source Activity

The source of theta activity during memory encoding under both randomized and organized conditions was localized by sLORETA. sLORETA is a standardized inverse solution that computes the three-dimensional cortical distribution of the source of neuronal activity from scalp EEG measurements to yield images of standardized current density with exact and zero-error localization (Pascual-Marqui, 2002). The computations of sLORETA were based on a realistic head model (Fuchs et al., 2002) using the Montreal Neurological Institute (MNI) 152 template (Mazziotta et al., 2001), with the three-dimensional solution space restricted to cortical gray matter, as determined by the probabilistic Talairach atlas (Lancaster et al., 2000). The intracerebral volume was partitioned in 6239 voxels (voxel size: 5 mm × 5 mm × 5 mm). sLORETA images represented the standardized electric activity at each voxel in neuroanatomic MNI space as the exact magnitude of the estimated current density. Anatomical labels as Brodmann areas were reported using MNI space, with correction to Talairach space (Brett et al., 2002).

Standardized low resolution brain electromagnetic tomography has been validated as an accurate estimation of the potential sources of the scalp EEG (Thatcher et al., 2005b; Bai et al., 2007; Dumpelmann et al., 2012), and that spatial accuracy of sLORETA can be maintained with as few as 19 electrodes given the specific signal to noise properties of the sLORETA algorithm (Pascual-Marqui, 2002; Pascual-Marqui et al., 2002). There were a number of studies that verified the reliability and validity of sLORETA for EEG source localization. For instance, De Ridder et al. (2011) have found consistent results between sLORETA source localized signals and fMRI brain activation pattern in the anterior and posterior cingulate cortex after alcohol craving suppression. Cannon and Baldwin (2012) have also reported that, comparing to the fMRI results, sLORETA was reliable for examining the default mode network and could localize to 5 mm^3^. In addition, another study conducted by Cannon et al. (2011) has shown converging results of sLORETA and fMRI in adults with ADHD during a Stroop task, and concluded that sLORETA was adequate in localizing the EEG sources, as contrasted with fMRI, and that the sLORETA could provide important information about the direction of difference relative to the BOLD signal increase.

In the present study, the prefrontal and parietal cortices were selected as the ROIs based on repeated empirical evidence for their involvement in the executive control of memory processing (Weiss et al., 2000; Fletcher and Henson, 2001; Rugg et al., 2002; Nyberg et al., 2003; Osaka et al., 2004; Sauseng et al., 2005). Furthermore, the medial and inferior temporal cortex, a well-known area for episodic memory acquisition (Squire and Zola-Morgan, 1991; Squire, 1992; Dickerson and Eichenbaum, 2010), was another ROI for exploring any potential training-induced changes in theta source activity for each group of participants.

### Data Analyses

The mean learning scores and theta coherence values of each group in both the randomized and organized conditions of the visual memory test pre- and post-training were compared using repeated measures ANOVAs followed by *post hoc* paired *t-*tests. The corresponding changes in mean scores and coherence values in each group were also computed for between-group comparisons using ANOVA. The source of theta activity as localized by sLORETA was compared within groups using built-in voxel-by-voxel paired sample *t-*tests. Because specific hypotheses were tested, no adjustment of the alpha level was applied for the planned comparisons to avoid lowering the power of the tests.

## Results

### NGT Enhances Performance and Memory Retrieval Strategy in Individuals with ASD

At the baseline, the three groups of participants demonstrated comparable performances in the three memory measures (total recall, semantic clustering, and visual scanning) under both randomized and organized conditions [*F*(2,45) = 0.09 to 5.79, *p* ≥ 0.006; i.e., the adjusted alpha level for multiple comparisons]. Given that their baseline memory performance was not associated with their age and diagnosis (*r* ranges from -0.03 to 0.24, *p*s > 0.05), the effects of age and diagnosis will not be taken into account in subsequent analyses. To compare the pre- and post-training changes in the three measures across groups under each condition, a separate 2 × 3 (time by group) repeated measures ANOVA was performed. There was a significant time by group interaction effect in semantic clustering scores [*F*(2,45) = 4.29, *p* = 0.02] in the randomized condition and in total recall [*F*(2,45) = 5.38, *p* = 0.01] and visual scanning scores in the organized condition [*F*(2,45) = 3.38, *p* = 0.04]. The results of a *post hoc* paired *t*-test suggested that the significant interaction effect resulted from the significant improvement in those measures in children who received NGT (*t* ranges from 2.44 to 3.11, *p* ranges from 0.03 to 0.006, effect size ranges from 0.57 to 0.73). There were no differences in the other two groups (*t* ranges from 0.41 to 1.59, *p* ranges from 0.69 to 0.13, effect size ranges from 0.11 to 0.39). Furthermore, there was a trend toward increasing total recall in the randomized condition after NGT (*t* = -1.27, *p* = 0.22), although the difference was not significant. This change was 3–8 times greater than that of the other two groups. Results of Pearson correlation analysis showed a significant negative correlation between the IQ score and the pre–post difference score in visual scanning at organized condition (*r* = -0.43, *p* = 0.04), and a trend of negative correlation between the IQ score and the pre–post difference score in total recall at both conditions (*r* = -0.33 and -0.34, *p* = 0.09). The results suggested that children with lower level of intellectual functioning tended to benefit more from the NGT with greater extent of improvement in memory performance. Furthermore, the extent of improvement of memory performance was not associated with their duration of *Nei Gong* practice throughout the study period (*r* ranges from -0.16 to 0.23, *p*s > 0.1). The lack of association is to be expected due to the fact that the duration of practice was not fixed and the children were instructed to practice with a natural and relaxed manner and cease doing the exercise when they began to sweat to avoid exhaustion. Given that the physical conditions varied among children, a longer practicing time would not necessarily result in greater memory improvement.

**FIGURE 2:**
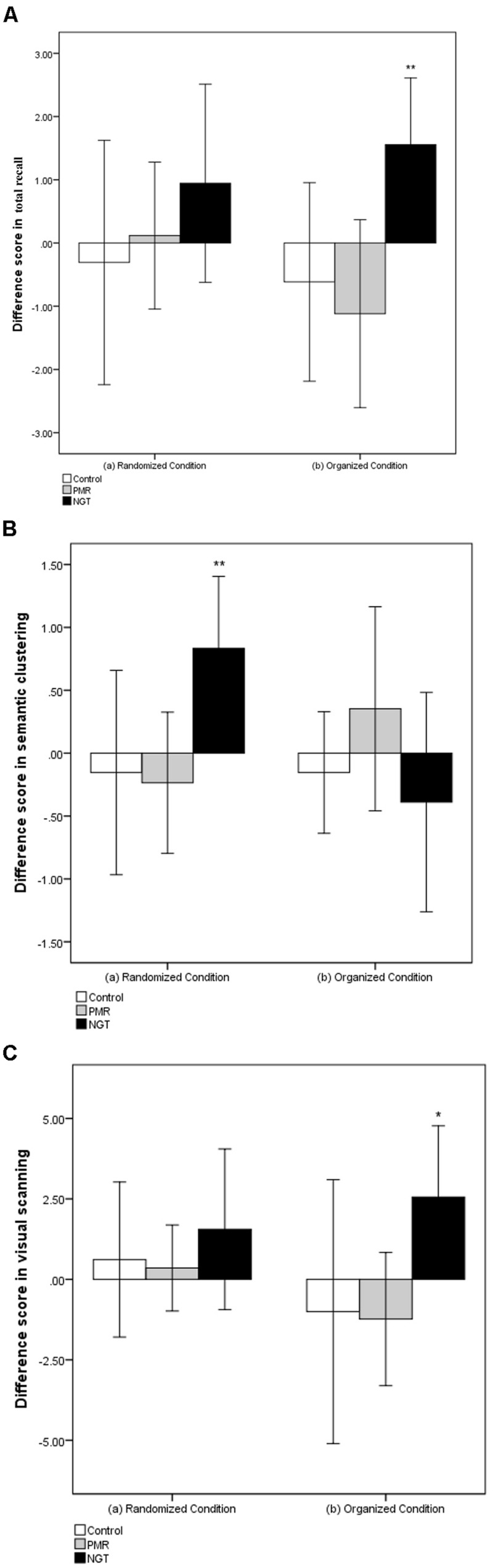
**Post-training score minus pre-training score in **(A)** total recall, **(B)** semantic clustering, and **(C)** visual scanning in randomized and organized conditions.** Positive values indicate improved performance. The error bars represent 95% confidence interval of the difference between pre- and post-training scores. Control, control group without training; PMR, progressive muscle relaxation; NGT, *Nei Gong* training. ^∗^*p* < 0.05; ^∗∗^*p* < 0.01 (paired samples *t*-test).

The difference in pre- and post-training scores is presented in **Figure 2**. Visual inspection of the pattern of changes in **Figure 2** suggests that NGT facilitated a positive change in memory recall and retrieval strategy in both the randomized and organized conditions, while the other two groups either demonstrated a decline in scores or less improvement. Further between-group comparisons were performed to compare the extent of changes across groups. Children with NGT showed greater improvement in semantic clustering scores in the randomized condition [*F*(2,45) = 4.29, *p* = 0.02] and in total recall [*F*(2,45) = 5.38, *p* = 0.008] and visual scanning scores [*F*(2,45) = 3.38, *p* = 0.04] in the organized condition compared with children receiving PMR practice or no training.

Overall, regular practice of *Nei Gong* for 1 month helped enhance memory recall and memory retrieval strategies. The extent of enhancement had a medium effect size. In contrast, children who received PMR training or no training did not demonstrate any improvement in memory recall and retrieval strategies.

### NGT Enhances Frontoposterior Theta Coherence during Memory Encoding in ASD

The baseline levels of theta coherence acquired from the frontal and posterior scalp regions were comparable among the three groups under both conditions (*F* range, 0.32–2.01; *p* range, 0.15–0.73). For the frontoposterior coherence values, the control group was higher than the NGT and PMR groups (*F* range, 2.76–5.69; *p* range, 0.01–0.074). Tukey *post hoc* tests showed that the NGT and PMR groups were comparable, with *p* values ranging from 0.60 to 1.00. The *p* values for the differences between the NGT and control groups ranged from 0.02 to 0.18, and those for the differences between the PMR and control groups ranged from 0.02 to 0.10. Separate 2 × 3 (time by group) repeated measures ANOVAs were performed to compare pre- and post-training changes in theta coherence among the three groups for each intra- and inter-hemispheric coherence value. There were no significant time by group interaction effects (*F* range, 0.003–2.67; *p* range, 0.08–1.00). There was a significant main effect of time or group in all intra- and inter-hemispheric frontoposterior coherence values (*F* range, 3.67–4.79; *p* < 0.05), except for the intra-right frontoposterior coherence in the randomized condition. Further within-group differences were compared across groups in subsequent *post hoc t*-tests.

**FIGURE 3:**
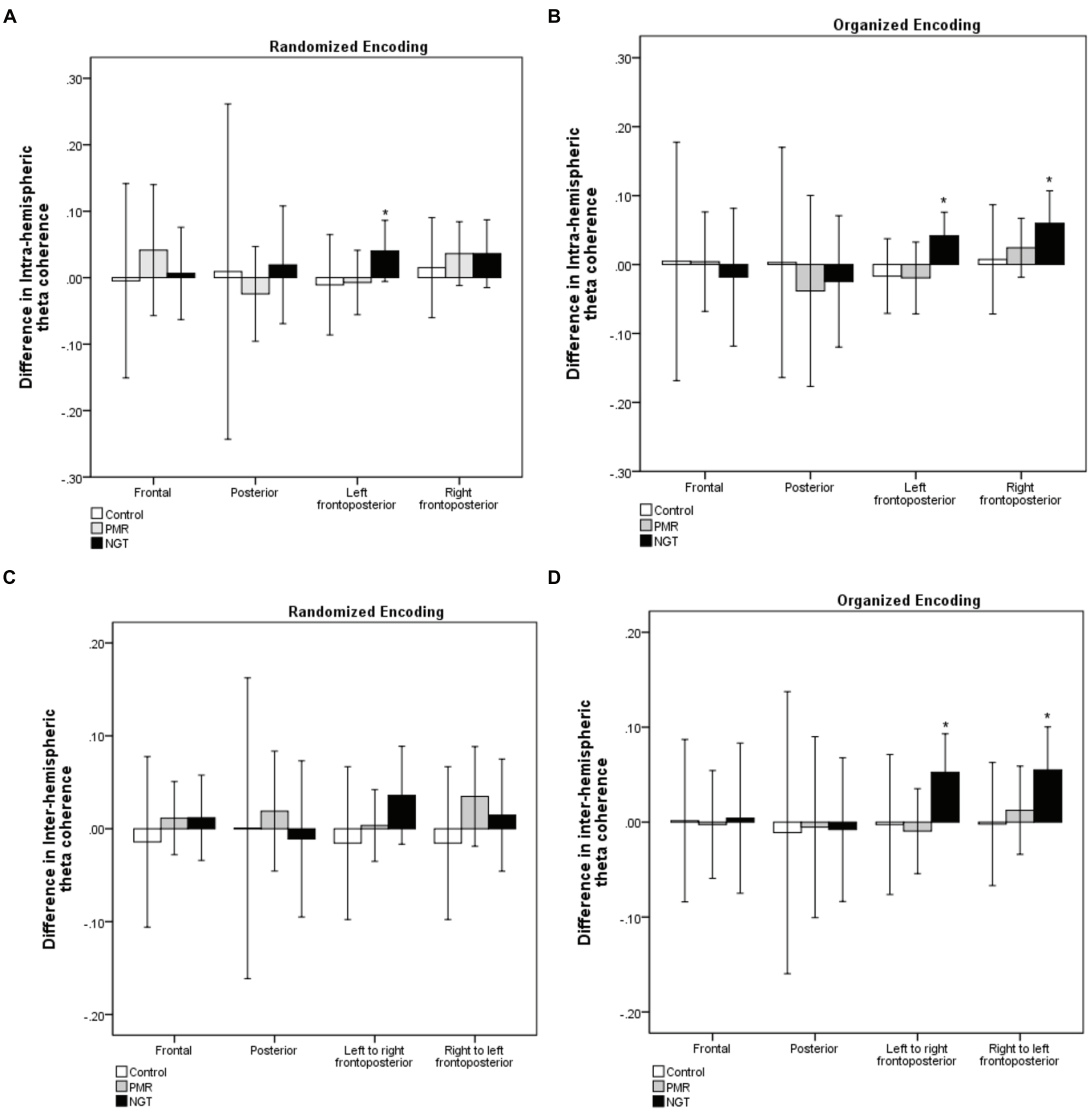
**Changes in **(A,B)** intra-hemispheric and **(C,D)** inter-hemispheric theta coherence after 1 month in the three groups in the randomized and organized conditions.** Positive values indicate increased theta coherence. The error bars represent 95% confidence interval of the difference between pre- and post-training theta coherence values. Control, control group without training; PMR, progressive muscle relaxation; NGT, *Nei Gong* training. ^∗^*p* < 0.05 (paired samples *t-*test).

**Figure 3** presents the changes in intra- and inter-hemispheric coherence values in the randomized and organized conditions. The results of paired samples *t*-tests suggested that children with NGT showed significantly increased theta coherence in certain frontoposterior connections, including intra-left frontoposterior coherence (*t* = 1.85, *p* = 0.04) in the randomized condition and intra-left and intra-right frontoposterior coherence (*t* = 2.58 and 2.67, *p* = 0.02 and 0.01) and inter-left-to-right and inter-right-to-left frontoposterior coherence (*t* = 2.74 and 2.57, *p* = 0.01) in the organized condition. In contrast, there were no significant changes in intra- and inter-hemispheric frontal or posterior coherence across groups (*p* > 0.05). Further analysis using Pearson correlation was attempted to explore the association of changes in frontoposterior coherence with level of intellectual functioning and with practice duration, if any. It was found that there was neither significant correlation between coherence changes and level of functioning (*r* ranges from -0.13 to 0.19, *p*s > 0.05), nor that between coherence changes and practice duration (*r* ranges from -0.02 to 0.21, *p*s > 0.20).

**FIGURE 4:**
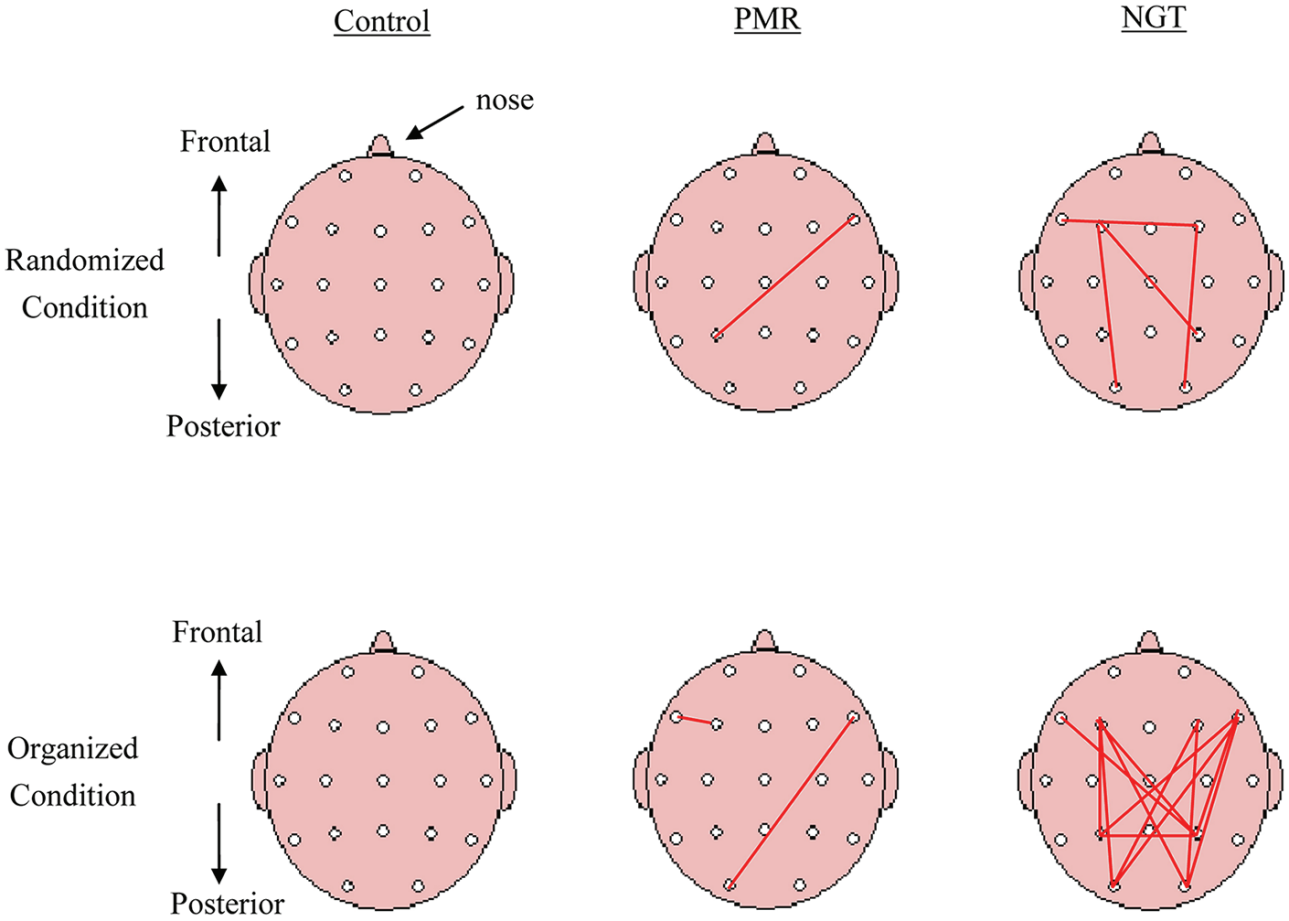
**Line topographic maps demonstrate changes in individual theta coherence pairs after training in the randomized and organized conditions across the three groups.** Red lines represent coherence pairs showing a significant increase in theta coherence (*p* < 0.05, paired samples *t*-test). Control, control group without training; PMR, progressive muscle relaxation; NGT, *Nei Gong* training.

The line topographic maps in **Figure 4** illustrate the 28 individual coherence pairs between the eight regions of electrode connections. Dots on the scalp represent the placement of the electrodes. Red lines connecting electrodes indicate electrode pairs that yielded a significant elevation in theta coherence after 1 month as analyzed by paired *t*-tests (*p* < 0.05). Consistent with the comparisons of mean coherence values, children with NGT showed greater elevation in theta coherence, including long-range connections between frontal and posterior scalp regions, while the other groups did not show a substantial change in coherence levels after 1 month. Compared with the randomized condition, there was a more pronounced increase in frontoposterior coherence values, covering 85% of possible electrode pairs, after NGT in the organized condition. In contrast, the PMR group only showed elevated coherence in 15% of electrode pairs, and the control group did not show any significant change in coherence.

### NGT Enhances Theta Source Activity in the Neural Network Underlying Memory Encoding in Individuals with ASD

**FIGURE 5:**
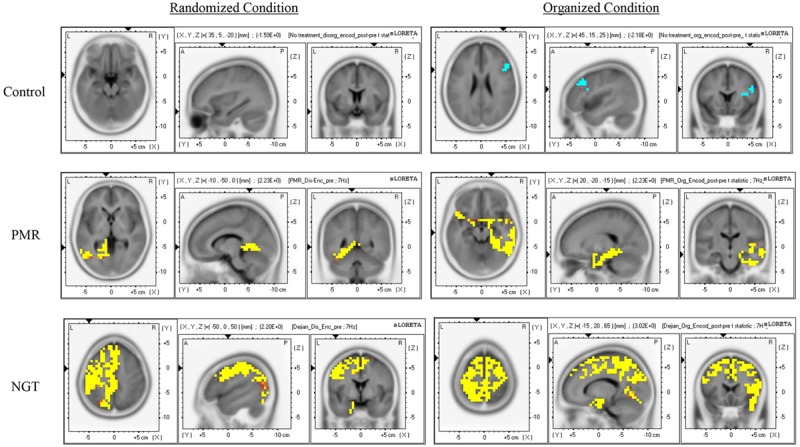
**Graphical representation of the sLORETA paired *t*-statistics results comparing the pre- and post-training theta source activity of the three groups in the randomized and organized conditions.** The regions colored in yellow/red indicate significantly elevated activity after training, while those colored in blue indicate significantly reduced activity, *p <* 0.05. Control, control group without training; PMR, progressive muscle relaxation; NGT, *Nei Gong* training.

Next, we localized the source of theta activity by sLORETA to explore the underlying neural network mediating the neural activity change in memory encoding due to training. The comparisons were focused on the pre–post source activity change at the ROIs (prefrontal cortex, parietal cortex, and temporal cortex) using sLORETA voxel-by-voxel paired *t* statistics for each group. After 1 month of NGT, the children showed significantly elevated theta source activity in the bilateral prefrontal cortex, the left parietal cortex, and the medial and inferior temporal cortex in the randomized condition (*t* maximum = 2.18; *p* < 0.05; **Figure 5**). This pattern of elevated source activity was more pronounced and bilateral during the organized condition (*t* maximum = 3.02; *p* < 0.05). The number of significant voxels found in the prefrontal cortex, the parietal cortex and the medial and inferior temporal cortex during the organized condition was 2.8, 2.4, and 16.8 times greater than during the randomized condition, respectively (**Table 3**). In order to examine the data consistency, further examination on the change in current density values of each individual at the corresponding MNI coordinates yielding the maximal *t* value based on group analysis result was performed. It was found that the individual data were generally consistent with the group-based data. There were 72 and 78% of participants in the NGT group demonstrated an increase in current density during randomized and organized conditions respectively, which is significantly more than those having a reduced current density, χ^2^ = 3.56 and 5.56, *p* = 0.03 and 0.009.

**Table 3:** Cortical voxels showing significant post-training changes in theta source activity across three groups and two memory conditions.

Memory condition	Group	Anatomical region	Brodmann areas (BAs)	No. of Sig. Voxels		Max. *t*-value
				Left	Right	
Randomized	Control	Prefrontal cortex	-	-	-	-
		Parietal cortex	-	-	-	-
		Medial and inferior temporal cortex	-	-	-	-
	PMR	Prefrontal cortex	-	-	-	-
		Parietal cortex	-	-	-	-
		Medial and inferior temporal cortex	19, 20, 27, 30, 36, 37	67	-	2.23
	NGT	Prefrontal cortex	6, 8, 9, 32, 44	253	99	2.18
		Parietal cortex	5, 7, 39, 40	88	-	1.95
		Medial and inferior temporal cortex	19, 28, 34, 35, 37	14	-	1.91
Organized	Control	Prefrontal cortex	8, 9, 44, 45, 46	1	28	–2.18
		Parietal cortex	-	-	-	-
		Medial and inferior temporal cortex	-	-	-	-
	PMR	Prefrontal cortex	9, 47	6	9	1.87
		Parietal cortex	-	-	-	-
		Medial and inferior temporal cortex	19, 20, 21, 27, 28, 30, 34, 35, 36, 37	14	237	2.23
	NGT	Prefrontal cortex	6, 8, 9, 10, 11, 13, 25, 32, 44, 45, 46, 47	361	638	3.02
		Parietal cortex	5, 7, 39, 40	201	10	2.46
		Medial and inferior temporal cortex	19, 20, 21, 27, 28, 30, 34, 35, 36, 37	57	178	2.37

*No. of Sig. Voxels = number of voxels in the left and the right hemisphere of the cortex with significant changes in current density above the corresponding *t* threshold value (^∗^*p* < 0.05). Max. *t*-value = the highest *t*-value within a specific anatomical region, indicating the greatest significant increase (positive value) or decrease (negative value) in theta source activity. Control, control group without training; PMR, progressive muscle relaxation; NGT, *Nei Gong* training.*

There was also an increased right-hemisphere involvement during the organized condition. The more widespread activation may be suggestive of a training-induced stimulation of the semantic network during the organized condition. In support of it, empirical evidence has shown that cognitive processing involved in the manipulation of semantic knowledge would acquire a more extensive brain networks consisting of the frontal, temporal and parietal cortices in healthy individuals (Binder et al., 2009; Simanova et al., 2014), whereas findings from previous study assessing the effect of behavioral treatment have demonstrated the enhanced memory performance was associated with increased cerebral activation in patients with multiple sclerosis (Chiaravalloti et al., 2012). Therefore, it is reasonable to assume that effective memory training, modulated by the effect of *Nei Gong* practice, should involve increased activations between multiple brain areas, and that the category cues provided in the organized condition might have activated the semantic network during information encoding in the children with autism. In addition, our findings are also consistent with previous study that the more extensive brain activation pattern may be suggestive of a greater balance between excitation of relevant information and inhibition of unwanted distraction (e.g., Chan et al., 2013b). It should be noted that there was also involvement of the inferior and medial frontal gyri (BAs 10, 11, 25, 32), which play a salient role in inhibitory control, during the organized but not the randomized condition. The findings are consistent with previous studies that suggested that the inhibitory deficit associated with frontal lobe dysfunction may underlie the memory impairment in ASD, in that individuals with ASD were found to be vulnerable to interference and produced significantly more false alarm responses during memory tasks (Cheung et al., 2010). On the contrary, the current density increase in the prefrontal cortex was lateralized to the left side during the randomized condition. This result is sensible in the sense that left prefrontal cortex has been reported to be largely involved in semantically related processing and its activity increases with learning strategies that require greater demand on semantic analysis (e.g., Gabrieli et al., 1998). During the randomized condition, the NGT group demonstrated greater improvement in semantic clustering, suggesting that the children in the NGT group were more apt in categorizing the objects during memory encoding, and that the improved semantic clustering in the NGT group was associated with greater activation of the left prefrontal cortex as suggested by the sLORTEA results.

Children who received PMR training did not show as robust a pattern of change as the NGT group. During the randomized condition, the PMR group only demonstrated a significant increase in source activity at the left medial and inferior temporal cortex (*t* maximum = 2.23; *p* < 0.05; **Figure 5**). The increased activity in the temporal cortex may facilitate memory encoding. The absence of changes in prefrontal and parietal activity may suggest a lack of change in cognitive processes for associating or analyzing stored semantic knowledge during memory encoding or in allocating and maintaining visuospatial attention. Although there was additional involvement of the prefrontal cortex during the organized condition, it was minimal compared with NGT group (**Table 3**). Further examination of the individual data of current density values was generally consistent with the group analysis results, showing relatively less robust increase in current density at the corresponding MNI coordinate yielding maximal *t* value. It was found that 69 and 63% of participants in PMR group demonstrated increased current density values during randomized and organized condition respectively, which is greater than those having a reduced current density though non-significantly, χ^2^ = 2.25 and 1.00, *p* = 0.07 and 0.16. Children who did not receive any training showed no activity change during the randomized condition and a significant reduction in prefrontal cortex activity during the organized condition (**Figure 5**).

## Discussion

The present study explored the potential neurocognitive enhancement effect of a mind–body exercise, *Nei Gong*, on memory function in children with ASD. We also examined the possible impact of *Nei Gong* on altering the neural mechanisms underlying memory processing using functional connectivity measures in children with ASD. Our findings suggest that children who received neurocognitive enhancement by *Nei Gong* for 1 month demonstrated enhanced memory retrieval, as reflected by their increased total recall, and enhanced memory retrieval strategies, as reflected by their increased semantic clustering and visual scanning scores. No significant improvement was observed in memory processing for the PMR and no-training groups.

After NGT, children appeared more capable of adopting different strategies to assist their memory, which may suggest better executive control of memory processing. When to-be-learned material was randomly presented to them, they appeared to be more aware of the semantic relationships among the material, as indicated by their higher tendency to recall information in clusters corresponding to semantic categories. This resulted in increased scores of memory recall. This tendency to categorize is self-generated because there was no category cue provided for the children during testing. Furthermore, in the organized condition, the items were pre-organized according to their semantic category during the encoding phase; i.e., items belonging to the same category were placed in the same row of the array. In this condition, the placement of the items was an additional cue for memory. Children with NGT largely relied on the visuospatial cues provided in the array during memory retrieval, as suggested by their elevated scores in visual scanning and their decreased trend for semantic clustering. Such a shift in strategy appears to indicate that the children became more flexible in problem solving. This shift was effective, as their correct hits were further enhanced in the organized condition. In contrast, children in the PMR and the no-training groups appeared to remain unaware of the semantic relationships among items and/or were unable to initiate the use of this strategy to assist memory. The *Nei Gong*-specific enhancement in memory performance and memory strategies was consistent with our previous findings of the positive effects of *Nei Gong* on memory and executive functions in patients with various brain disorders (Chan et al., 2011d, 2012, 2013a,b, 2014a) and community elderly with lower memory functions (Chan et al., 2014b). Furthermore, the effect of *Nei Gong* on memory-enhancement was in moderate size (0.57–0.73), which is compatible to that of tDCS studies reporting increased memory performance on tasks that required effortful learning (Cohen’s *d* = 0.6 in Marshall et al., 2004; Cohen’s *d* = -0.5 and 0.4 in Hammer et al., 2011). Given the findings of improved memory performance and more flexible adoption of learning strategy in children who have practiced *Nei Gong*, it is conceivable that NGT might exert a knock-on effect on other cognitive performance, such as academic performance or progress in other forms of treatment. Therefore, further study with measurement on those aspects is warranted.

More interestingly, the memory enhancement after NGT correlated with an increased synchronization of EEG signals between frontal and posterior brain regions, as reflected by elevated EEG coherence indices, suggesting the improved memory performance in the NGT group may be related to enhanced functional network that mediates the executive control of memory process. This finding is consistent with previous studies reporting the involvement of the prefrontal-parietal loop in effective memory encoding and retrieval during tasks that involved manipulation rather than simple retrieval and maintenance of information (Weiss et al., 2000; Sauseng et al., 2005). Furthermore, sLORETA current density localized the source of scalp EEG signals to the ROIs, including the prefrontal, the parietal and the temporal cortex. The involved ROIs also coincide with the target brain regions (prefrontal and temporoparietal cortices) where tDCS or TMS has been applied to improve memory functions of healthy adults and patients with memory disorders (Marshall et al., 2004; Solé-Padullés et al., 2006; Boggio et al., 2009, 2012; Hammer et al., 2011; Coffman et al., 2014). Again, this alteration in functional connectivity of the neural system was not observed in the other two groups of children. The present study has replicated our previous EEG findings on the effect of *Nei Gong* on altering neural mechanism underlying higher-order cognitive processes, including memory, inhibitory control and cognitive flexibility (Chan et al., 2011a, 2012, 2013a,b). It has also provided further support for the potential neurocognitive enhancement effect of *Nei Gong* in fostering functional reorganization of central nervous system connectivity to promote higher levels of brain efficiency and more effective use of strategies to monitor, organize and maintain information during memory tasks. The idea that NGT results in a more efficient brain is sensible, as our previous RCT already reported a positive effect of *Nei Gong* on fostering a state of mind that can facilitate peak performance (i.e., the co-existence of a relaxed and an attentive mind; Chan et al., 2011a).

Our findings are also consistent with previous neuroimaging and neuropsychological studies reporting that the effects of other forms of mind–body training on higher-order cognitive processing are associated with underlying functional changes within the brain (Davidson et al., 2003; Newberg et al., 2010; Tang et al., 2010; Hasenkamp and Barsalou, 2012; Xu et al., 2014). Functional imaging studies assessing the effects of meditation, a form of mind–body training, have demonstrated significantly increased activation of executive control of memory and attention networks. In a SPECT study conducted by Newberg et al. (2010), they demonstrated a significant increase in memory recall that converged with an increase in cerebral blood flow in the prefrontal and parietal cortices in 15 adults having memory loss after an 8-weeks meditation program. In addition to the elevated activity in the prefrontal and parietal cortices, higher brain activation in the medial temporal lobe that is crucial for memory retrieval has been found in experienced practitioners of non-directive meditation techniques (Xu et al., 2014). Similarly, brain imaging studies have also reported alteration in the frontoparietal network associated with focused and sustained attention and error monitoring in meditators relative to age-, years of education-, and sex-matched controls (Kozasa et al., 2008; Short et al., 2010; Hasenkamp and Barsalou, 2012). These research findings thus provide support to the notion that mind–body training activates structures involved in higher-order cognitive processes and leads to enhanced functional connectivity (Kilpatrick et al., 2011) throughout the entire brain. This connectivity, in turn, leads to enhanced levels of brain efficiency and improved cognitive function (Kozasa et al., 2012).

Our study showed that for children with ASD, practicing *Nei Gong* for approximately 20–30 min per day for 1 month enhanced learning capacity and enabled children to adopt more effective strategies during a memory task. Our electrophysiological findings demonstrated that NGT increased theta coherence and theta activation over cortical areas known to be involved in learning and memory. Thus, converging evidence suggests the possibility of clinical application of mind–body exercise, as a neurocognitive enhancement technique, for enhancing memory functions in children with ASD. Nevertheless, the generalization of our findings to the wider ASD patient population may be limited due to our relatively small sample size, the over-representation of high-functioning children with ASD, the wide variation in ages, and random training effect confounded by the experience and skills of the therapists providing the training regimens. Future randomized controlled studies using the same therapist to provide the different training regimens, recruiting a larger sample size to compare different age groups, observe the treatment effects across time with longitudinal follow-up, and including low-functioning individuals with ASD and different clinical populations would help extend the current findings to a wider population. In addition, it would be interesting to investigate whether the treatment effects of NGT can be generalized beyond the context of visual memory and exert further knock-on effects on improving school performance and problem behaviors. While our findings demonstrated the effects of 1 month of NGT, its long-term effects remain unclear. Thus, future investigation is warranted to explore its effect at longer term. Furthermore, to delineate the therapeutic effects of NGT, it would also be interesting to compare the NGT to other forms of well-studied mind–body training technique, such as *Yoga* and *Tai Chi*.

## Conclusion

The present study has revealed potential effects of a Chinese mind–body exercise, *Nei Gong*, on facilitating more flexible and effective use of strategies to aid memory in children with ASD. The memory-enhancement effect converged with the alteration of functional connectivity in the neural system as measured by EEG coherence and localized by the sLORETA method, suggesting that children with ASD demonstrated the application of a more efficient neural network for memory processing after the practice of *Nei Gong*. These encouraging findings have provided insight into the clinical applicability of *Nei Gong* as a possible neurocognitive enhancement approach for individuals with ASD.

## Author Contributions

AC, SS, and EL have contributed to the conceptualization of the study and the design of experiment. All authors have involvement in acquisition, analysis or interpretation of data, preparation of the manuscript, final approval of the manuscript, and ensuring that questions related to the accuracy or integrity of the study are appropriately investigated and resolved.

## Conflict of Interest Statement

The authors declare that the research was conducted in the absence of any commercial or financial relationships that could be construed as a potential conflict of interest.

## Abbreviations

ASD: autism spectrum disorders
NGT: *Nei Gong* training
PMR: progressive muscle relaxation
ROIs: regions of interest
